# Mini-laparoscopic cholecystectomy: evolution of a new technique

**DOI:** 10.1186/s12893-021-01389-8

**Published:** 2021-11-03

**Authors:** Ali Warsi, Andrew Natsuki Wilson, Kin Seng Tong, Jonathan Gan, Ho Lun Chong

**Affiliations:** 1 General Surgery, University Hospitals of Morecambe Bay NHS Foundation, Lancaster, UK; 2 Department of Surgery and Cancer, Imperial College London, London, UK; 3 23 Nightingale House, Pointer Court, Lancaster, LA1 4JT UK

**Keywords:** General surgery, Gastrointestinal surgery, Pancreas and biliary tract, Cholecystectomy

## Abstract

**Background:**

Since the first laparoscopic cholecystectomy (LC) in 1985, there has been much advancement in laparoscopic surgery in terms of reduction in number and size of ports. We report a new technique of performing mini laparoscopic cholecystectomy using only three ports, 5 mm each. The indications of this procedure include GB polyps, GB dyskinesia, microlithiasis, and idiopathic pancreatitis.

**Case presentation:**

In this case report, we present a new technique that has been performed safely in a 49-year-old male patient with pancreatitis caused by microlithiasis. This was performed using a novel three port procedure consisting of only 5 mm ports, and he was discharged as a day case without complications. Informed patient consent was obtained.

**Conclusions:**

The fundamentals of this mini-LC technique remain the same as that of a standard laparoscopic cholecystectomy throughout the procedure. It is a feasible option in selected cases, and it has the potential to further augment the inherent benefits of minimal access surgery namely less analgesia, improved cosmesis and faster recovery. Further trials will help ascertain its potential advantages.

## Background

Since the first laparoscopic cholecystectomy (LC) performed by Muhe in 1985 [1], there are several modifications in its technique in terms of reductions in number and size of ports. The conventional or standard technique consists of four port LC—10 mm × 2 and 5 mm × 2 ports. There are now many modified or new techniques to this conventional four-port technique, such as three-port or single incision surgery [1, 2]. Port sizes have also reduced from the standard 10 mm trocar to 5 mm, 3 mm or 2 mm [3]. Due to the numerous possible combinations of reduced port size and/or number in laparoscopic cholecystectomy, different names have been given to these modifications, such as ‘mini-laparoscopic cholecystectomy’, ‘micro-laparoscopic cholecystectomy’ and ‘needloscopic surgery’, with various definitions [4–6]. However, they have retained the use of a 10 mm port. We report a new modified technique of performing LC using only 5 mm ports × 3 (3 ports of 5 mm diameter), which has not been reported before.

## Case presentation

A 49-year-old man was admitted with epigastric pain. History suggestive of acute pancreatitis was confirmed on routine investigations with a raised amylase of 1040. Investigations to elicit the common aetiology of acute pancreatitis were normal: absence of gallstones (GS) on ultrasound (USS), lack of history of alcohol intake, normal lipids and calcium. He was not on any regular medications. He was readmitted again over the course of the next 3 months with similar presentation and raised amylase but normal USS and again normal liver function tests. His gastroscopy and repeat USS of the abdomen were normal. A subsequent endoscopic ultrasound revealed microlithiasis, a recognised cause for pancreatitis [7]. The gall bladder status was Parkland grading scale Grade 1 [8]. He was subsequently listed for LC. This was performed using a novel three port procedure consisting of only 5 mm ports, and he was discharged as a day case without complications. Informed patient consent was obtained.

**Fig. 1:**
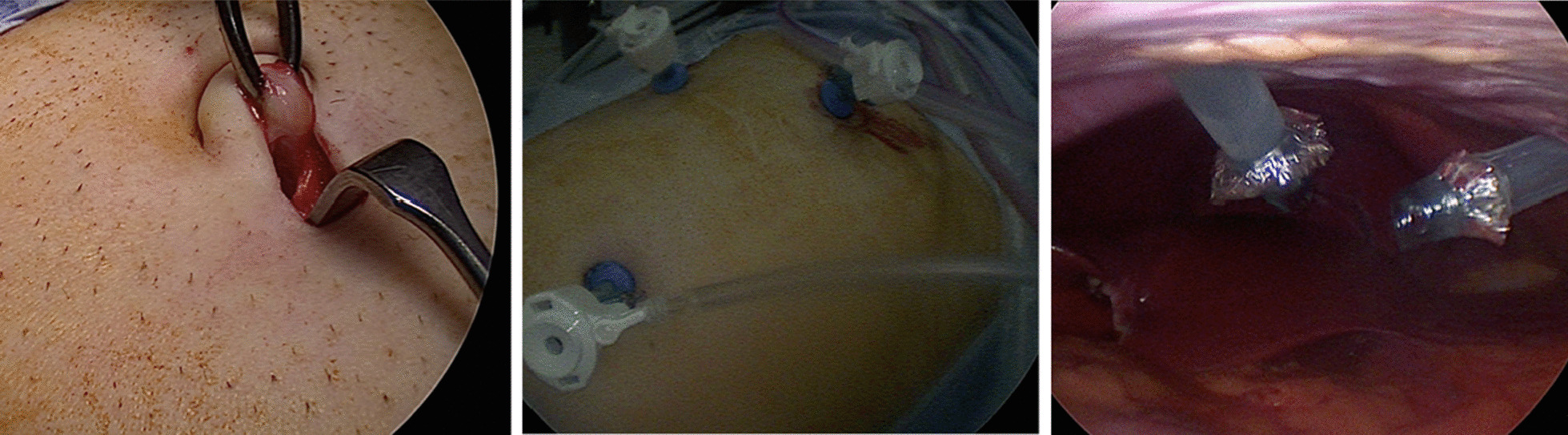
(Left to right) Creation of 5 mm umbilical port, Port placement during surgery, Laparoscopic view during surgery

**Fig. 2:**
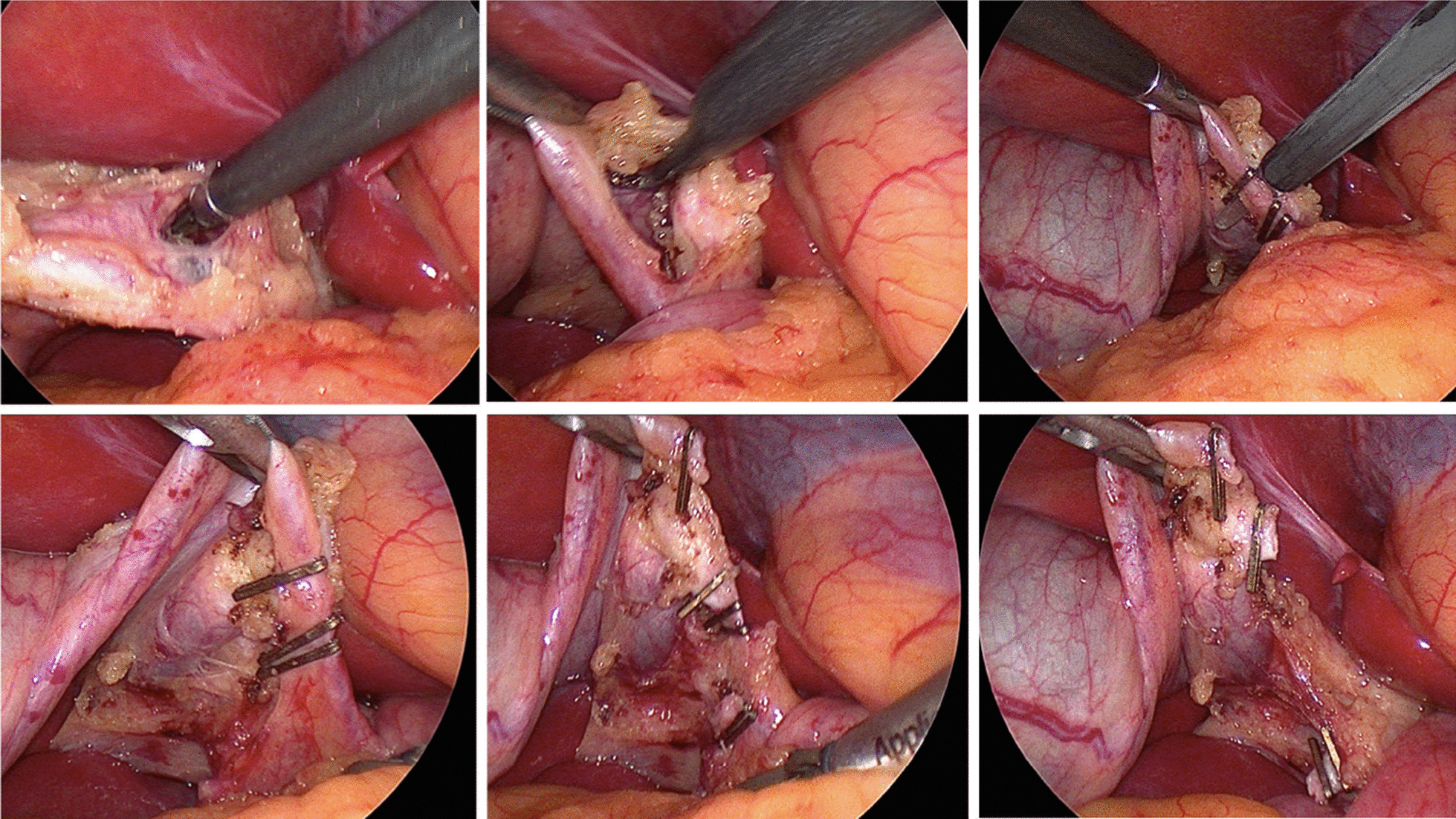
(Upper row left to right) Demonstration and dissection of Calot’s triangle, clipping of cystic duct of GB. (Bottom row left to right) Demonstration of critical view, clipping and cutting of cystic duct and cystic artery

**Fig. 3:**
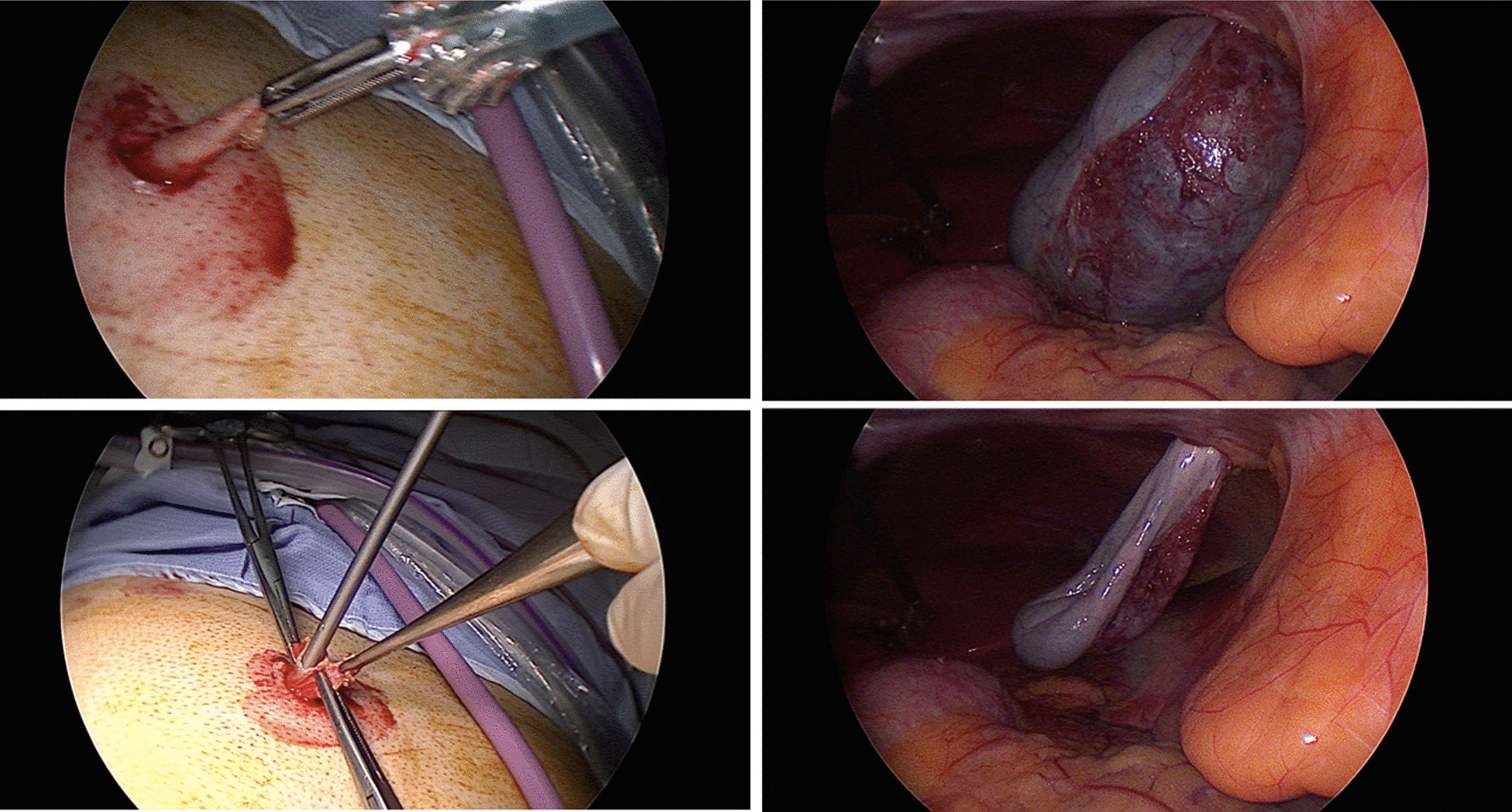
(Upper row left to right) GB delivered out through the abdominal wall after cholecystectomy, inside view of drawing GB into the epigastric port. (Bottom row left to right) Suction and aspiration of the GB with a laparoscopic sucker, leaving a shrivelled GB

**Fig. 4:**
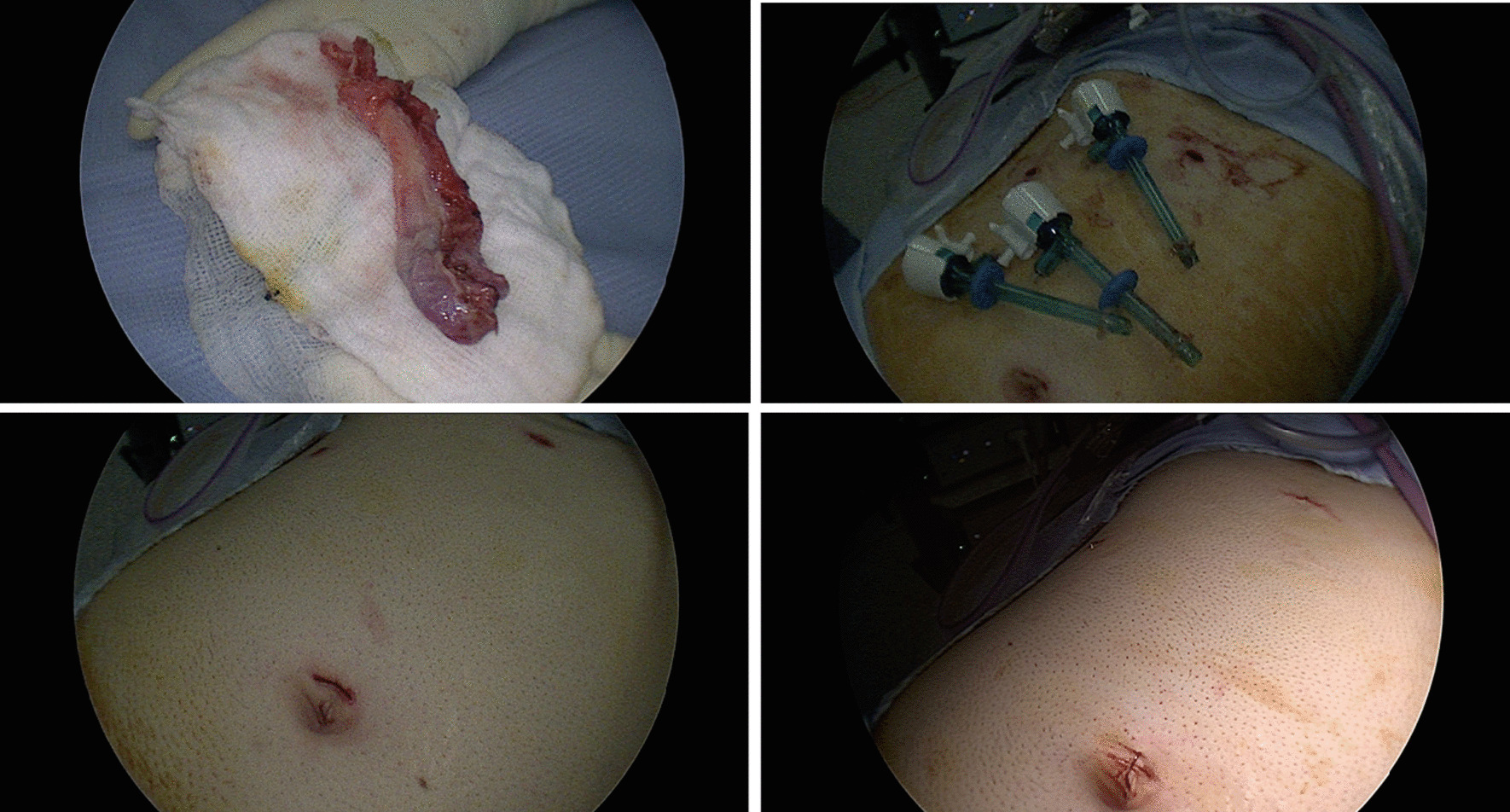
(Upper row left to right) GB specimen. End of surgery and ports removed. (Bottom row left to right) Post surgery scar and clips

The surgical technique is fundamentally similar to a standard LC and is demonstrated in Figs. 1, 2, 3 and 4. Standard disposable 5 mm balloon ports × 3 (applied sciences) were placed in supraumbilical, epigastric and RUQ (in midclavicular line). The standard 4th port in the anterior axillary line was not inserted in this case. We used a high quality 5 mm camera in the umbilical port. The other two ports each 5 mm were inserted under direct vision. Patient was in reverse Trendelenburg position with right side of the patient tilted to the left by approximately 30°. A Maryland forceps and diathermy hook was used via the epigastric port and a Johans forceps via the RUQ port. The gallbladder (GB) was grasped at a convenient point just above the Hartmann’s pouch and retracted upwards and outwards or downwards as required to gain exposure to the Calot’s triangle. A Maryland forceps and hook connected to diathermy was used to grasp and strip or divide the peritoneum and create windows on the lateral side and medial sides of the Calot’s triangle staying close to the GB. Sufficient length of cystic duct and artery were displayed, clipped with a 5 mm laparoscopic clipper-2 proximally and 1 distally and divided. The GB was dissected off the liver bed. Through the 5 mm epigastric port the GB was grasped with a Johans forceps near its cystic duct end and withdrawn into the port. The port was gently withdrawn such that the distal GB and the distended fundus was snugly stuck into the epigastric port and the proximal end protruding from the abdominal wall. A small incision between two clips to this exposed part of the GB allowed suction and aspiration of the GB with a laparoscopic sucker to leave a shrivelled G.B. This was easily coaxed out, withdrawn and sent for histology. The epigastric port was reinserted to carry out a final inspection of the operative field and help with infiltration of Marcaine-20 mls of 0.25% marcaine to the GB fossa and 20 mls of 0.5% to the three ports. Ports were removed under direct vision checking that there was no bleeding or oozing. ‘J’ Needle Vicryl ‘1’ suture was used to close the 5 mm umbilical port and skin was closed with single staple to be removed in 8 days and Mepore dressing applied. This patient was discharged with uneventful postoperative recovery as a day case.

## Discussion and conclusion

We present the first reported case of a three port 5 mm only mini LC. This technique has evolved and has been standardised over the course of last 5–10 years. The author has experience of assisting and performing three port LC previously. However, this included two ports of 10 mm and 1 of 5 mm, and this technique has previously been reported [9]. With the availability of 5 mm good quality camera and a 5 mm laparoscopic clip applier the author was able to perform three port LC with a 10 mm × 1 and 5 mm ports × 2. The author then realised that for selected cases, a 10 mm port may not be necessary to extract the gall bladder. Hence 5 mm × 4 port LC was performed in 2015 which at the time was not previously reported. It was then felt that the need for the 4th port or the lateral most port in the anterior axillary line might not be necessary. On 16th July 2016, the novel mini LC needing only 3, 5 mm laparoscopic ports was performed. Subsequently we have performed 10 cases with 3 or 4 port 5 mm LC with good outcomes. The indications to perform this procedure in selected cases are GB polyps, GB dyskinesia, microlithiasis, and cases of pancreatitis where no cause has been identified. The gall bladder status of these cases range from Parkland grading scale Grades 1–2 [8]. Regarding cases involving large gallstones, enlargement of the retrieval port may still be required.

The basic fundamentals of the laparoscopic cholecystectomy procedure as per the Tokyo guidelines in 2018 remain unchanged [10]. The GB is appropriately retracted to develop a plane in the Calot’s triangle area and allow for its boundaries to be identified. Dissection is then started from the posterior leaf of the peritoneum covering the neck of the GB to expose the GB surface above Rouviere’s sulcus. The plane of dissection of the GB surface is maintained throughout the procedure. Dissection of the lower part of the GB bed (at least one third) is performed to obtain a ‘critical view of safety’ of the Calot’s triangle. This critical view is maintained before clipping and dividing both the cystic duct and cystic artery. Given that these principles stay the same in this procedure and there is no change in fundamental methods technique associated with LC, this procedure does not need to be notified to the medical governance committee as advised by the National Institute of Clinical Excellence (NICE) new interventional procedures guidelines (2009) [11].

The various nomenclature and approaches to mini LC has been very well summarised in the review article by Haribhakti et al. [12]. Performing laparoscopic cholecystectomy with reduced port number and/or size brings its own technical challenges; for instance, the vision achieved with a 5 m camera is limited compared to that with a 10 mm one. Nonetheless, a good view of Calot’s triangle is still possible in patients with a short GB and a floppy liver [12]. Since the 5 mm camera image quality is not as good as that of a 10 mm, it is of utmost importance that a good quality 5 mm camera be used so as not to compromise the dissection and division of cystic duct and artery and avoid damage to other structures namely the common bile duct (CBD). Moreover, it is possible to facilitate visualisation of the Calot’s triangle by fundal traction, achieved via a suture inserted from the right lower chest wall [12]. In our experience, we had been able to perform mini-laparoscopic cholecystectomies successfully without requiring fundal traction in selected patients for the indications discussed earlier. Besides, using our gallbladder retrieval technique, we did not need to dilate or increase the size of the incision in any of our mini LC cases, hence preserving the 5 mm scars post operatively.

Mini-laparoscopic cholecystectomy offers numerous potential advantages. In terms of reduced port number, Trichak randomised 200 consecutive patients undergoing elective laparoscopic cholecystectomy to be treated either with a three- or four-port technique, and demonstrated that the former resulted in less pain, lower cost and fewer scars [9]. Al-Azawi et al. undertook a retrospective review of 495 patients receiving either three-port or four-port laparoscopic cholecystectomies and found that the three-port procedure was associated with less pain and a shorter hospital stay [13]. Mayir et al. showed that the three-port approach was comparable to the four-port approach in terms of operation time, length of stay in hospital, complication rate, and rate of conversion to open surgery [14]. With regards to reduced port size, Novitsky et al. reported decreased early post-operative incisional pain and superior cosmetic results in 79 elective laparoscopic cholecystectomies performed using 10 mm umbilical, 5 mm epigastric, 2 mm subcostal and 2 mm lateral ports [5]. Furthermore, a retrospective review by McCormack et al. of 79 patients who had undergone elective laparoscopic cholecystectomies via a 5 mm trocar for the umbilical port and 3 mm trocars for other ports demonstrated good results with no conversion to open surgery nor intra- or post-operative complications [6]. It is notable that in their study, an endocatch bag was used to deliver the GB out through the 5 mm port, which is different from the technique described in our paper. Shaikh et al. compared the use of the standard four trocars (10 mm × 2, 5 mm × 2) with mini-laparoscopic cholecystectomy using three 3 mm ports and one 10 mm port, and found that the latter was not only comparable to the standard ports in terms of blood loss, post-operative pain, analgesia requirement and mobilisation, but was also associated with earlier return to work and superior cosmetic outcomes [15].

In conclusion, our novel mini LC involving only three 5 mm ports is an feasible option in selected cases, converting to the four-port in difficult cases if required so as not to compromise safety. It has the potential to augment the inherent benefits of minimal access surgery. Further trials will help ascertain its potential advantages.

## Data Availability

The data that support the findings of this study are available from the corresponding author, upon reasonable request.

## Abbreviations

LC: Laparoscopic cholecystectomy
GB: Gallbladder
CBD: Common bile duct
GS: Gallstones
USS: Ultrasound
NICE: National Institute of Clinical Excellence
